# The adenosine A2A receptor in human sperm: its role in sperm motility and association with *in vitro* fertilization outcomes

**DOI:** 10.3389/fendo.2024.1410370

**Published:** 2024-05-30

**Authors:** Houyang Chen, Genbao Xing, Wenqing Xu, Ying Chen, Leizhen Xia, Hua Huang, Jialv Huang, Qing Hong, Tao Luo, Hao Wang, Qiongfang Wu

**Affiliations:** ^1^ Reproductive Medical Center, Jiangxi Maternal and Child Health Hospital, Nanchang, Jiangxi, China; ^2^ Jiangxi Key Laboratory of Reproductive Health, Nanchang, China; ^3^ Institute of Biomedical Innovation, Jiangxi Medical College, Nanchang University, Nanchang, Jiangxi, China; ^4^ Department of Urology, The First Affiliated Hospital, Hengyang Medical School, University of South China, Hengyang, Hunan, China

**Keywords:** adenosine A2A receptor, sperm motility, *in vitro* fertilization, intracellular calcium concentration, CatSper

## Abstract

**Background:**

The involvement of ATP and cAMP in sperm function has been extensively documented, but the understanding of the role of adenosine and adenosine receptors remains incomplete. This study aimed to examine the presence of adenosine A2A receptor (A2AR) and study the functional role of A2AR in human sperm.

**Methods:**

The presence and localization of A2AR in human sperm were examined by western blotting and immunofluorescence assays. The functional role of A2AR in sperm was assessed by incubating human sperm with an A2AR agonist (regadenoson) and an A2AR antagonist (SCH58261). The sperm level of A2AR was examined by western blotting in normozoospermic and asthenozoospermic men to evaluate the association of A2AR with sperm motility and *in vitro* fertilization (IVF) outcomes.

**Results:**

A2AR with a molecular weight of 43 kDa was detected in the tail of human sperm. SCH58261 decreased the motility, penetration ability, intracellular Ca^2+^ concentration, and CatSper current of human sperm. Although regadenoson did not affect these sperm parameters, it alleviated the adverse effects of SCH58261 on these parameters. In addition, the mean level of A2AR in sperm from asthenozoospermic men was lower than that in sperm from normozoospermic men. The sperm level of A2AR was positively correlated with progressive motility. Furthermore, the fertilization rate during IVF was lower in men with decreased sperm level of A2AR than in men with normal sperm level of A2AR.

**Conclusions:**

These results indicate that A2AR is important for human sperm motility and is associated with IVF outcome.

## 1 Introduction

During the process of fertilization, the primary mechanism by which sperm achieve motility and fertilization is through the stimulation of flagellar movement, which requires a significant expenditure of energy. This energy is predominantly derived from the hydrolysis of adenosine triphosphate (ATP) generated during sperm metabolism (1). Furthermore, the adenosine cyclase/cyclic adenosine monophosphate (AC/cAMP) system plays a crucial role in both flagellar oscillation and sperm capacitation (2). Adenosine, a vital component in the synthesis of ATP and cAMP, is thus indispensable for the proper functioning of sperm (3). Adenosine, acting as the primary ligand for sperm cells, interacts with adenosine receptors (ARs) located on the cell membrane and serves as the endogenous ligand for all adenosine receptors (4). ARs are classified within the G-protein coupled receptor superfamily, with each receptor exhibiting both activation and inhibition effects (5). Research has demonstrated the presence of two distinct histidine residues within the active site of ARs, one serving as the binding site for agonists and the other serving as the binding site for inhibitors (6). The AR system in mammals is categorized into three subtypes: A1, A2, and A3 receptors. Within the A2 subtype, there are further divisions into A2A and A2B receptors based on the affinity between adenosine and the receptors. Notably, adenosine A2 receptor (A2R) has been found to have significant implications for the development and progression of various clinical diseases (7, 8).

Several studies have highlighted the role of adenosine in regulating sperm function through its interaction with A2Rs. Fraser et al. conducted mouse sperm studies and reported that adenosine and its analogs can activate AC via A2Rs, leading to the promotion of intracellular cAMP synthesis. This, in turn, mediates the regulation of sperm capacitation and fertilization ability in mice (9). Additionally, Shen et al. further validated the impact of adenosine on sperm motility through A2Rs present on the sperm membrane (10). Romac et al. conducted a study to confirm the role of adenosine in stimulating human sperm motility and activating dynamin ATPase through A2Rs using adenosine and its analogs (11). The aforementioned research indicates that A2Rs play a significant role in regulating sperm function. However, the specific mechanisms by which A2AR affect sperm motility and their potential correlation with sperm motility and clinical success in assisted reproductive techniques, such as *in vitro* fertilization (IVF), remain unclear. Further investigation is required to explore these aspects.

Therefore, in this study, the presence and localization of A2AR were examined in human sperm by western blotting and immunofluorescence assays. The functional role of A2AR in sperm was assessed by incubating human sperm with an A2AR agonist (regadenoson) and antagonist (SCH58261). The sperm level of A2AR was examined by western blotting in normozoospermic and asthenozoospermic men to evaluate the association of A2AR with sperm motility and IVF outcomes. Our results may provide new insights into the function of A2AR in human sperm.

## 2 Materials and methods

### 2.1 Human sperm collection

Semen samples were obtained through the process of masturbation from individuals who were receiving care at the Reproductive Medical Center of Jiangxi Maternal and Child Health Hospital in Nanchang, China, following a period of abstinence lasting 3 to 5 days. This study was performed in accordance with the guidelines outlined in the World Health Organization Laboratory Manual for the Examination and Processing of Human Semen (5th edition). In total, 80 normozoospermic men who had normal sperm quality and 89 asthenozoospermic men (with a sperm count >15 million cells/mL, viability >58%, progressive motility <32%, and total motility <40%) were recruited for this study. The semen characteristics of the participants are shown in **Supplementary Table S1**. The studies involving human semen samples were approved by the ethics committee of Jiangxi Maternal and Child Health Hospital (Nanchang, China). The studies were conducted in accordance with local legislation and institutional requirements. The participants provided written informed consent to participate in this study.

### 2.2 Immunofluorescence analysis

Immunofluorescence assays were performed according to our previous study (12). Fresh human sperm were collected and fixed with 4% paraformaldehyde (Solarbio, Beijing, China) for 10 min. The fixed sperm were applied to the bottom of confocal dishes (Nest Biotechnology Co., Ltd., Wuxi, China) pretreated with 1% poly-L-lysine (Solarbio, Beijing, China) for 20 min. Then, the sperm were permeabilized with 0.2% Triton X100 (Solarbio, Beijing, China) in phosphate-buffered saline (PBS) (Solarbio, Beijing, China) for 15 min and subsequently blocked with 10% normal goat serum (NGS; Solarbio, Beijing, China) in PBS containing 0.05% Triton X-100 for at least 90 min at room temperature. An anti-A2AR antibody (Abcam ab288412, USA; 1:100) was incubated overnight at 4 °C with 5% NGS in PBS containing 0.05% Triton X-100. DyLight 488-labeled goat anti-rabbit IgG (EarthOx, San Francisco, CA, USA; 1:100) was used as the secondary antibody. Rabbit IgG (Solarbio, Beijing, China; 1:200) was used as a negative control. The immunofluorescence-stained sperm were imaged using a Nikon super-resolution structured illumination microscope (SIM) (Nikon Co. Ltd., Tokyo, Japan). The images were captured with a Nikon ECLIPSE Ti-E inverted microscope using a CFI Apochromat TIRF 100× oil objective lens (N.A. 1.49). The experiment was repeated at least three times.

### 2.3 Western blotting

According to our previous paper, total proteins were isolated from sperm of 80 normozoospermic and 89 asthenozoospermic men (13). Total proteins (30 µg) were used for western blotting as previously described (13). The dilutions used for the primary antibodies were as follows: anti-adenosine receptor A2A antibody (1:500) and anti-beta actin antibody (1:10000). The dilutions used for the secondary antibodies were 1:10000 for the HRP-conjugated goat anti-rabbit secondary antibody. The membranes were visualized using an enhanced chemiluminescence (ECL) detection kit (PK10001; Proteintech, China) according to the manufacturer’s instructions.

### 2.4 Measurement of sperm motility

The normozoospermic sperm were harvested by direct swim-up in human tubal fluid (HTF). The sperm were treated with 1 μM regadenoson (MedChemExpress LLC, Monmouth Junction, NJ, USA), 10 μM SCH58261 (MedChemExpress LLC), or 1 μM regadenoson plus 10 μM SCH58261 in HTF for 4 h at 37 °C in a 5% CO_2_ incubator. Total motility and progressive motility were evaluated by Hamilton Thorne CEROSII computer assisted sperm analysis (Hamilton Thorne Biosciences, Beverly, MA, USA). At least 200 sperm were counted in each trial.

### 2.5 Measurement of penetration ability

The normozoospermic sperm were harvested by direct swim-up in HTF. The sperm were treated with 1 μM regadenoson, 10 μM SCH58261, or 1 μM regadenoson plus 10 μM SCH58261 in HTF plus NaHCO_3_ and human serum albumin (HTF++) for 4 h at 37 °C in a 5% CO_2_ incubator. Penetration ability was analyzed by evaluating the ability of sperm to penetrate a 1% (w/v) methylcellulose (Sigma Chemical Co., St. Louis, MO, USA) solution prepared in HTF++ as described previously (14).

### 2.6 Measurement of the intracellular Ca^2+^ concentration

The sperm were loaded with 5 µM Fluo-4 AM (Thermo Fisher Scientific, Waltham, MA, USA) and 0.05% Pluronic F-127 (Thermo Fisher Scientific) in the dark at room temperature for 30 min and then washed in HTF. Washed sperm were placed in microplates (Thermo Fisher Scientific), and Fluo-4 fluorescence was detected using an EnSpire® multimode plate apparatus (PerkinElmer Inc., Waltham, MA, USA). The sperm were recorded for 80 s before the addition of 1 μM regadenoson, 10 μM SCH58261, 1 μM regadenoson+10 μM SCH58261 or equivalent HTF, and the data were recorded for 300 s after administration. The change in sperm [Ca^2+^]_i_ was calculated by ΔF/F_0_ (%), which represents the change in fluorescence (ΔF) normalized to the percentage (%) of average basal fluorescence before any chemical (F_0_) was applied. F/F_0_ (%) = (F−F_0_)/F_0_× 100%.

### 2.7 Sperm patch clamp recording

As previously described, the whole-cell patch-clamp technique was used to record CatSper current (14). A 20-30 MΩ pipette was used to seal the sperm cytoplasmic droplets in the HS solution. The switch to whole-cell mode is then achieved by applying a short (1 ms) voltage pulse (400-650 mV) combined with light suction. The current is stimulated by a voltage ramp of 1 s, from −100 to +100 mV, with a holding potential of 0 mV. The basic CatSper univalent current was recorded using a divalent free (DVF) solution (150 mM NaCl, 20 mM HEPES, 5 mM EDTA, pH 7.4). Then, the CatSper current was evaluated after infusion of 1 μM regadenoson, 10 μM SCH58261, or 1 μM regadenoson+10 μM SCH58261. The data were analyzed using Clampfit software (v10.4, Axon, Gilze, Netherlands).

### 2.8 Grouping method for IVF analysis

The inclusion criteria for women were as follows: aged 20-38 years, infertility caused by simple tubular factors, aged 22-42 years, and underwent conventional insemination with more than 4 eggs. Patients eligible for inclusion were divided into two groups according to the A2AR content: a control group with normal A2AR (36 patients) and a group with decreased A2AR (44 patients) according to the lowest sperm level of A2AR in normozoospermic men according to western blotting.

### 2.9 Statistical analysis

All the statistical analyses were performed with the statistical software GraphPad Prism (version 5.01, GraphPad Software, San Diego, CA, USA). The data are expressed as the mean ± standard error of the mean, and normally distributed data were analyzed by the Shapiro–Wilk test (p > 0.05). Differences between the A2AR levels in men with normozoospermia and asthenozoospermia were assessed using an unpaired t test, and linear regression was performed with GraphPad Prism. Statistically significant differences were determined at p < 0.05.

## 3 Results

### 3.1 A2AR is located in the human sperm tail

**Figure 1:**
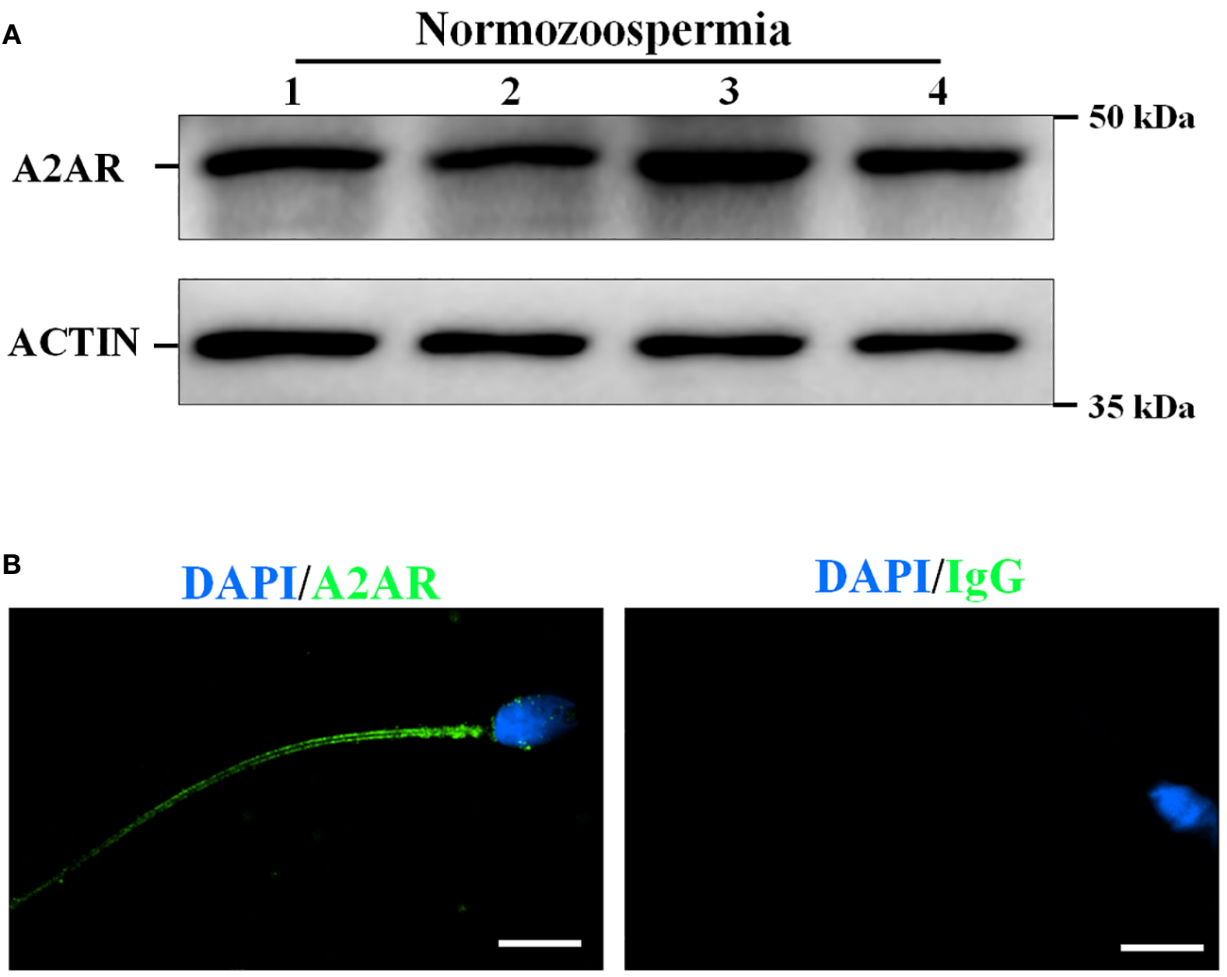
The presence and localization of adenosine A2A receptors (A2AR) in human sperm. **(A)** The proteins were isolated form four normozoospermic sperm (N) and the presence of A2AR was examined by western blotting. **(B)** The localization of A2AR was examined by immunofluorescence staining. Rabbit IgG as a negative control. Bar, 5 μm.

Western blotting analysis of freshly collected human sperm protein to further confirm the presence of A2AR in human sperm. Target bands between 37 and 50 kDa were clearly observed, and the predicted size of A2AR was 43 kDa (**Figure 1A**). In addition, immunolocalization of freshly collected sperm was performed by confocal microscopy analysis to reveal the presence of A2AR in human sperm. Immunofluorescence results showed that A2AR was located throughout the sperm tail (**Figure 1B**).

### 3.2 A2AR is involved in the regulation of human sperm motility

**Figure 2:**
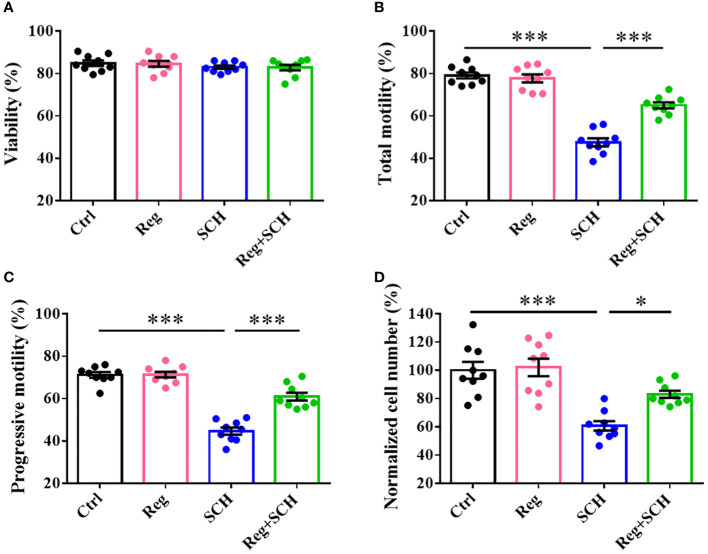
Effects of A2AR agonist and antagonist on human sperm motility. Human sperm were incubated with 1 μM Regadenoson, 10 μM SCH58261, and 1 μM Regadenoson+10 μM SCH58261 in human tubal fluid (HTF) or HTF plus NaHCO_3_ and human serum albumin (HTF++) for 4 h at 37 °C in a 5% CO_2_ incubator. The sperm viability **(A)**, total motility **(B)**, progressive motility **(C)**, and penetration ability **(D)** were examined. The experiments were repeated on 9 individuals. The data are presented as the mean ± standard error of the mean. Differences between groups were assessed by one-way ANOVA and Tukey’s test. *p < 0.05 and ***p < 0.001.

Neither the A2AR agonist (regadenoson) and nor the antagonist (SCH58261) affected sperm viability (**Figure 2A**). SCH58261 decreased total motility (**Figure 2B**), progressive motility (**Figure 2C**), and penetration ability (**Figure 2D**). Although regadenoson did not affect these sperm parameters, it alleviated the adverse effects of SCH58261 on these parameters (**Figures 2B-D**). These results indicate that A2AR plays an important role in human sperm motility.

### 3.3 Inhibition of A2AR reduced the [Ca^2+^]_i_ in human sperm

**Figure 3:**
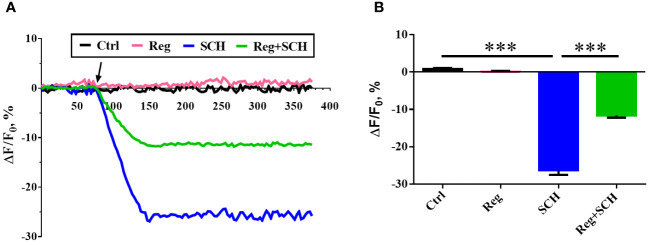
Effects of A2AR agonist and antagonis on sperm intracellular Ca^2+^ concnetration ([Ca^2+^]_i_). **(A)** Time course curve of real-time changes in human sperm [Ca^2+^]_i_. **(B)** Statistical analysis of the human sperm [Ca^2+^]i at the time point of 150 second. The experiments were repeated on 6 individuals. The data are presented as the mean ± standard error of the mean. Differences between groups were assessed by one-way ANOVA and Tukey’s test. ***p < 0.001.

The present study showed that the inhibition of A2AR by SCH58261 reduced human sperm motility and penetration ability. Since these processes are Ca^2+^ dependent, we conducted real-time monitoring of [Ca^2+^]_i_ in human sperm using Fluo-4 AM staining. SCH58261 decreased the human sperm [Ca^2+^]_i_ by approximately 26% after 70 s of administration and then continued to decrease until the end of the experiment (**Figures 3A, B**). Although regadenoson did not affect human sperm [Ca^2+^]_i_, it alleviated the adverse effect of SCH58261 on human sperm [Ca^2+^]_i_ (**Figures 3A, B**).

### 3.4 Inhibition of A2AR decreased the CatSper current

**Figure 4:**
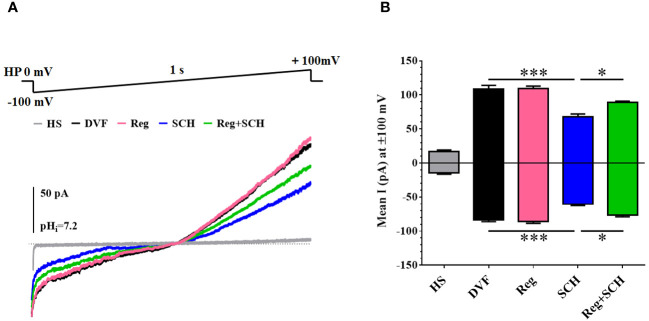
Effects of A2AR agonist and antagonis on Catsper currents. **(A)** Recording CatSper currents by whole-cell patch-clamp technique, using a ramp protocol of −100 to +100 mV. **(B)** Statistical analysis of the mean CatSper current at ±100 mV. The experiments were repeated on 6 individuals. The data are presented as the mean ± standard error of the mean. Differences between groups were assessed by one-way ANOVA and Tukey’s test. *p < 0.05 and ***p < 0.001.

To further determine the underlying mechanism of the SCH58261-induced decrease in the sperm [Ca^2+^]_i_, we examined the effect of SCH58261 on the current of CatSper, a predominant ion channel responsible for Ca^2+^ influx in human sperm. The basal CatSper current was recorded in a DVF solution. The mean currents at ± 100 mV measured in the DVF were decreased by SCH58261 from 109.0 pA (100 mV, DVF)/−83.3 pA (-100 mV, DVF) to 68.2 pA (100 mV, SCH)/−60.3 pA (−100 mV, SCH; **Figures 4A, B**). Although regadenoson did not affect the human CatSper current, it alleviated the adverse effect of SCH58261 on the human sperm CatSper current (**Figures 4A, B**).

### 3.5 The sperm level of A2AR is positively correlated with human sperm motility

**Figure 5:**
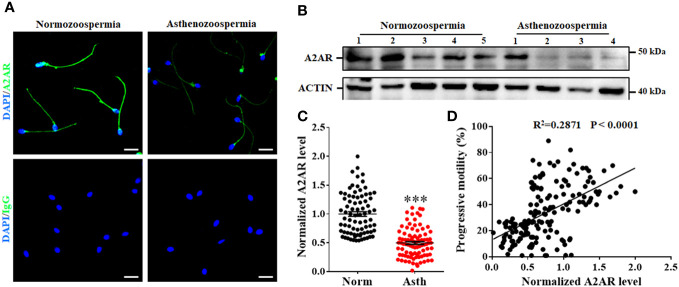
Relationship between A2AR and progressive motility of human sperm. Typical images of immunofluorescence **(A)** and western blotting **(B)** showed that sperm level of A2AR are generally lower in sperm from asthenozoospermic men (Asth) than those in normozoospermic men (Norm). Bar, 10 μm. **(C)** Semiquantification of A2AR in sperm from 80 Norm and 89 Asth. **(D)** The correlation between sperm level of A2AR and progressive motility was assessed by linear regression in 169 men. The data are presented as the mean ± standard error of the mean. Differences between groups were assessed by Student *t* test. ***p < 0.001.

To further verify the correlation between A2AR levels and sperm motility, we first compared the sperm level of A2AR between normozoospermic men and asthenozoospermic men. Typical examples of immunofluorescence analysis (**Figure 5A**) and western blotting (**Figure 5B**) showed that A2AR levels were generally lower in asthenozoospermic men than in normozoospermic men (**Figures 5A, B**). Quantification of the western blotting results revealed that the mean level of sperm A2AR was reduced by approximately 50% in asthenozoospermic men compared to normozoospermic men (**Figure 5C**). Linear regression analysis was performed to evaluate the relationship between the A2AR level and progressive motility. The results showed that the sperm level of A2AR was positively correlated with the progressive motility of human sperm (R^2^ = 0.2871, *p* < 0.0001; **Figure 5D**).

### 3.6 Relationship between sperm A2AR and clinical IVF outcomes

To further assess the relationship between sperm A2AR and IVF outcomes, we performed a retrospective analysis. Asthenozoospermic men who received IVF treatment were selected. In total, 80 asthenozoospermic men were included. According to the lowest sperm level (0.54, **Figure 5C**) of A2AR in normozoospermic men, the 80 asthenozoospermic samples were divided into the A2AR-normal group (>0.54) and the A2AR-reduced group (<0.54). The couples in the A2AR-reduced group had a lower 2PN fertilization rate than did those in the A2AR-normal group (77.85% vs 62.99%, **Table 1**). However, there was no difference in age, number of eggs, rate of high-quality embryos, rate of HCG positivity, or clinical pregnancy rate between these two groups (**Table 1**).

**Table 1:** The in vitro fertilization outcomes of the A2AR-normal and A2AR-reduced groups.

	A2AR-normal group	A2AR-reduced group	*p* value
Number	36	44	–
Female age	28.77±3.37	29.42±4.32	0.43
Male age	30.47±3.84	30.60±3.90	0.87
Number of eggs	12.47±5.44	13.56±5.27	0.34
2PN fertilization rate	77.85 % (355/456)	62.99 % (371/589)	<0.001***
High quality embryo rate	37.30 % (119/319)	34.79 % (127/365)	0.5446
HCG positive rate	70.00 % (21/30)	66.67 % (24/36)	0.245
Clinical pregnancy rate	66.67 % (20/30)	63.89 % (23/36)	0.4594

Data are expressed as the mean ±  standard deviation.

***p < 0.001.

## 4 Discussion

Adenosine receptors, specifically the A1, A2, and A3 receptor subtypes, have been identified and successfully cloned in mammals (15). Within the A2 receptor subtype, further categorization can be made into A2A and A2B receptors based on their affinity for adenosine (15). However, under normal physiological conditions, the concentration of adenosine is insufficient to activate A2B receptors. Only in pathological conditions such as inflammation, hypoxia, or ischemia, where the concentration of adenosine in tissue fluid increases, adenosine can exert its effects through A2B receptors (16). Hence, it is plausible that adenosine exerts its influence on physiological adenosine levels through the activation of the A2AR in the A2 receptor of sperm. This finding aligns with the aforementioned experimental outcomes. The presence of A2AR, which has a molecular weight of approximately 43 kDa, in human sperm was verified through western blotting analysis in this study. Additionally, an immunofluorescence assay revealed the localization of A2AR in the tail of human sperm. These findings establish a foundation for the involvement of adenosine A2AR in the regulation of sperm function.

Sattin and Rall were the first to highlight the role of adenosine in cellular regulation, particularly through its interaction with specific receptors on the cell surface (17). These adenosine receptors are classified within the G protein receptor superfamily, which enables agonists to modulate adenylate cyclase activity and facilitate cyclic adenylate production (18). However, notably, different adenosine receptors exert distinct effects on adenylate cyclase. A1 and A3 receptors predominantly associate with the Gi (inhibitory) protein, leading to the inhibition of adenylate cyclase and subsequent reduction in cAMP synthesis. Conversely, A2 receptors are coupled with Gs (stimulatory) proteins, thereby promoting cAMP generation (19). Previous studies have demonstrated the significance of the A2 receptor, a stimulatory G protein receptor, in the regulation of sperm function (9, 11, 20). In line with these previous investigations, our study revealed a correlation between A2AR and sperm motility, whereby the inhibition of intracellular calcium through the use of the inhibitor SCH58261 impeded sperm motility. Notably, the testis contains various voltage-gated calcium channels, with the CatSper ion channel being exclusively located in sperm and responsible for the detection of [Ca^2+^]_i_ currents (21). Hence, a more comprehensive analysis was conducted on Catsper channel currents, revealing that SCH58261 effectively hindered intracellular calcium levels by inhibiting the aforementioned currents. This observation aligns with the previously established functional role of adenosine receptors. Consequently, an in-depth exploration was undertaken to explore the association between adenosine A2AR and sperm progressive motility. Our investigation revealed a positive correlation between adenosine A2AR and progressive sperm motility, with a notable decrease observed in asthenozoospermic individuals. These findings underscore the significance of A2AR in the maintenance of normal sperm motility.

Following ejaculation, sperm are unable to penetrate the zona pellucida of the oocyte for fertilization until they have undergone processes such as sperm capacitation, sperm hyperactivation, and acrosome reactions. These transformations occur either within the female reproductive tract or in a controlled culture medium *in vitro* (22). The occurrence of these physiological changes is contingent upon the involvement of cAMP. By interacting with A2AR on the surface of sperm cells, adenosine activates AC and subsequently facilitates cAMP synthesis. Research has demonstrated that cAMP plays a crucial role in regulating sperm function through activation of the protein kinase A (PKA) pathway (23). It has been reported that PKA-dependent phosphorylation activates CatSper (24–27). Activation of the PKA pathway enhances sperm hyperactivation and facilitates sperm movement toward the egg (24). These sperm processes are closely related to CatSper-mediated Ca^2+^ influx (14). In this study, we found that inhibition of A2AR decreased the CatSper current and sperm motility of human sperm. Interestingly, our investigation demonstrated significantly lower sperm motility and fertilization rates in the group with reduced A2AR than in the normal control group. These results suggest that A2AR may couple with the Gs protein, promote cAMP generation by activating AC, stimulate PKA-dependent phosphorylation of the CatSper complex, and thereby maintain Ca^2+^ influx to activate sperm motility and other feralization-associated processes. The findings of this study may contribute to the development of a framework for assessing sperm quality to enhance the effectiveness of *in vitro* fertilization (IVF).

Additionally, the study revealed that while there was no statistically significant difference in the quality of embryos or clinical pregnancy rates between the two groups, patients with decreased A2AR exhibited poorer outcomes than did those in the normal control group. However, it is important to note that the lack of statistical significance may be attributed to the limited sample size. Hence, the present study is limited by a restricted sample size, necessitating the inclusion of a larger sample to ascertain the association between sperm A2AR and IVF outcome. Concurrently, our findings indicate that A2AR influences sperm motility and intracellular calcium levels, resulting in diminished success rates of IVF. Consequently, additional investigations are warranted to explore the potential impact of reduced A2AR levels on post-fertilization embryo development. According to a previous report, oocyte analysis following IVF revealed gradual penetration of the sperm tail into the ovum during fertilization, potentially influencing the movement of male prokaryotes within the ovum and contributing to embryo formation (28). However, the specific involvement of the sperm tail A2AR in this process remains uncertain. Consequently, the regulatory mechanism of A2AR in sperm and its association with the development of fertilized eggs and early embryos remain ambiguous, necessitating further investigation by researchers.

## 5 Conclusions

In this study, we revealed that A2AR is predominantly located in the sperm tail. A2AR likely regulates human sperm motility by modulating Ca^2+^ influx through the CatSper channel. The sperm level of A2AR is positively correlated with progressive motility and is associated with the fertilization rate of IVF. Collectively, these findings indicate the involvement of A2AR in regulating human sperm motility and function.

## Data availability statement

The raw data supporting the conclusions of this article will be made available by the authors, without undue reservation.

## Ethics statement

The studies involving humans were approved by the ethics committee of Jiangxi Maternal and Child Health Hospital (Nanchang, China). The studies were conducted in accordance with the local legislation and institutional requirements. The participants provided their written informed consent to participate in this study.

## Author contributions

HC: Writing – review & editing, Writing – original draft, Project administration, Methodology, Funding acquisition, Conceptualization. GX: Writing – review & editing, Resources, Methodology, Investigation. WX: Writing – original draft, Methodology. YC: Writing – review & editing, Methodology, Investigation. LX: Writing – review & editing, Investigation. HH: Writing – review & editing, Investigation, Resources. JH: Writing – review & editing, Resources. QH: Resources, Writing – review & editing. TL: Writing – original draft, Writing – review & editing. HW: Investigation, Writing – review & editing, Project administration, Conceptualization. QW: Writing – review & editing, Project administration, Funding acquisition, Data curation, Conceptualization.
