# Supplementary material for: The adenosine A2A receptor in human sperm: its role in sperm motility and association with in vitro fertilization outcomes
Source: Front Endocrinol (Lausanne). 2024 May 30;15:1410370. doi: 10.3389/fendo.2024.1410370 (PMC11169588; doi:10.3389/fendo.2024.1410370)
Supplement: Supplementary file 1 [file Table_1.docx]

| Parameters | Normozoospermic men (n=80) | Asthenozoospermic men (n=89) | Reference |
| --- | --- | --- | --- |
| Age | 28.9±3.7  (22.0-36.0) | 28.4±3.5  (20-35) | Reproductive age |
| Viability (%) | 79.6±7.2  (64.0-92.0) | 70.6±5.4  (58.0-83.0) | > 58 |
| Total motility (%) | 63.8±13.2  (40.9-88.5) | 25.7±10.2  (5.1-39.4) | > 40 |
| Progressive motility (%) | 51.6±12.8  (32.6-89.0) | 16.9±8.3  (1.0-31.7) | > 32 |
| Volume (mL) | 2.9±0.6  (1.6-5.2) | 2.9±0.7  (1.5-5.0) | >1.5 |
| Sperm count  (10^6^ cell/mL) | 75.3±42.1  (16.2-185.7) | 51.8±29.9  (15.2-139.7) | >15.0 |

Table S1. Semen characteristics of the participants

Data are expressed as the mean ±  standard deviation (minimum-maximum).
